# Our Journal

**Published:** 1854-03

**Authors:** 


OUR JOURNAL.—There is another announcement which we are happy to make, and we are quite certain that our readers will share in the pleasure we take in doing so. When, eight months ago, we began the work of originating the "*Iowa Medical Journal*," from a mingled sense of duty and necessity, our hopes were not quite so numerous as our forebodings. The enterprize was new, and its success doubtful; but our chief reliance was in the fact that we were quite willing—

"To labor and to wait."

But it seems that our thorough performance of the former duty, has rendered the latter less essential. In fact, our success has far exceeded even our best hopes. Our patronage seems to increase in a sort of "compound proportion." We are thankful, therefore, for the success we have received, and in consequence are enabled to state that *the Journal will be enlarged* at the beginning of the second volume. We intend to increase our limits as fast as our prosperity will admit, and to make the Journal, year after year, renewedly worthy of support and encouragement. We make the announcement thus early, that our friends throughout the State may be stimulated to renew their efforts in our behalf, in order that we may set out upon our second voyage with a prosperous breeze.

---

God and the doctor, we alike adore ;
Just on the brink of danger, not before ;
The danger passed, both are alike requited ;
God is forgotten, and the doctor slighted !

